# A global meta-analysis of gillnet bycatch of toothed whales: Mitigation measures and research gaps

**DOI:** 10.1016/j.isci.2024.111482

**Published:** 2024-11-26

**Authors:** Christian Sonne, Su Shiung Lam, Shin Ying Foong, Wan Adibah Wan Mahari, Nyuk Ling Ma, Michael S. Bank

**Affiliations:** 1 Aarhus University, Faculty of Technical Sciences, Department of Ecoscience, DK-4000 Roskilde, Denmark; 2 Sustainability Cluster, School of Engineering, University of Petroleum & Energy Studies, Dehradun, Uttarakhand 248007, India; 3 Higher Institution Centre of Excellence (HICoE), Institute of Tropical Aquaculture and Fisheries (AKUATROP), Universiti Malaysia Terengganu, Kuala Nerus 21030 Terengganu, Malaysia; 4 University Centre for Research and Development, Department of Chemistry, Chandigarh University, Gharuan, Mohali, Punjab, India; 5 BIOSES Research Interest Group, Faculty of Science & Marine Environment, Universiti Malaysia Terengganu, Kuala Nerus, Terengganu 21030, Malaysia; 6 Center for Global Health Research (CGHR), Saveetha Medical College, Saveetha Institute of Medical and Technical Sciences (SIMATS), Saveetha University, Chennai, India; 7 Institute of Marine Research, Bergen, Norway; 8 University of Massachusetts Amherst, Amherst, MA, USA

**Keywords:** Environmental science, Environmental monitoring, Environmental policy

## Abstract

Odontocetes are globally distributed and are foundational to the structure and function of marine food webs, and hence bycatch impacts from gillnet fishing need to be considered in the context of their conservation and population viability. Currently, global gillnet bycatch numbers are unknown yet are estimated to be the greatest in Asia, East Africa, and the west coasts of North and South America. Here we provide the first global meta-analyses of small- and large-scale gillnet bycatch estimates of odontocetes during 1990–2020, compiling population size, estimated gillnet bycatch, and conservation status in support of geographical and species-specific risk estimates. We estimate that annual gillnet bycatch is ∼50,000 from 1990 to 2020, and, combined with overfishing, pollution, and noise, it has been shown to be a serious threat to these long-lived and slow-reproducing species with heavy offspring investment. The global gillnet bycatch of odontocetes is a difficult challenge to address and mitigate and requires improved species and regional-based management strategies including collaborations between fishers, fisheries managers, marine mammal experts, and marine spatial planners. This has been worked on for decades, yet more attention is needed for successful management of odontocete gillnet bycatch to ensure their sustainable future in the Anthropocene Ocean, in accordance with local subsistence dynamics and the relevant United Nations (UN) sustainable development goals.

## Graphical abstract

**Figure:**
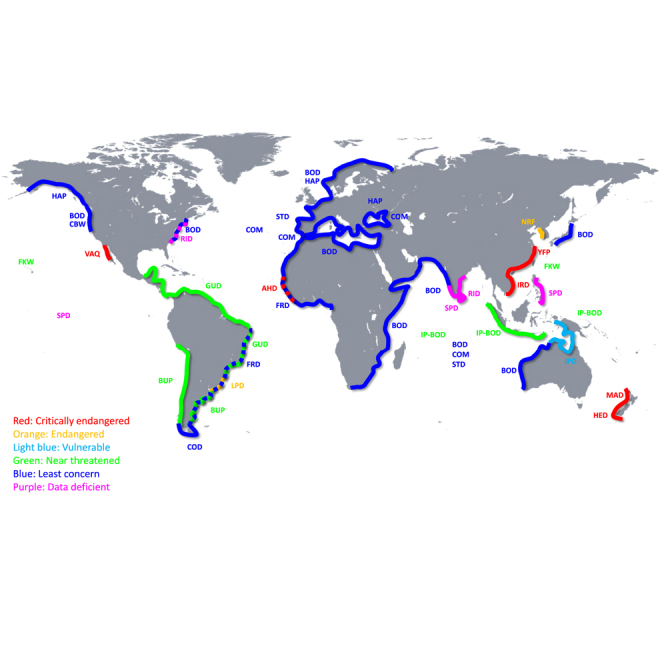

## Introduction

**Figure 1:**
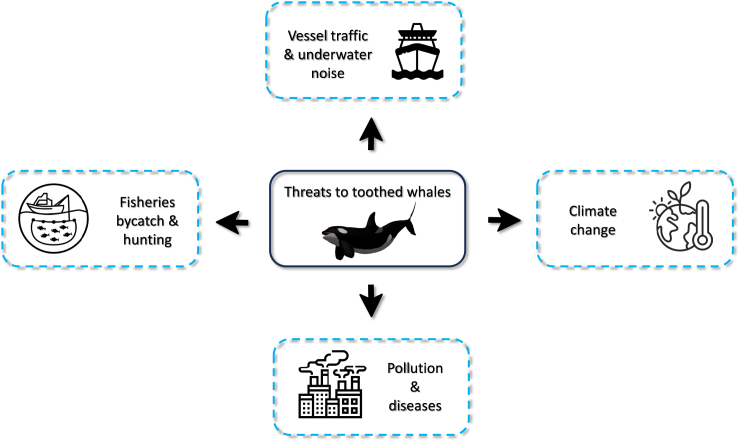
Current threats to odontocetes worldwide from increasing human activities including fisheries, hunting, gillnet bycatch, vessel traffic, underwater noise, pollution, climate change, and disease

Toothed whales (odontocetes), including dolphins and porpoises, are found worldwide and require carefully structured management efforts to safeguard their populations. These species currently face large historical and anthropogenic impacts, including fisheries and gillnet bycatch, overharvesting, ocean pollution, and underwater noise (Figure 1).^1^ These species are of immense socioeconomic and ecological value, play a vital role in maintaining food web structure and function, contribute to essential ecosystem services, and support thriving eco-tourism industries.^2^^,^^3^^,^^4^^,^^5^ Marine mammals are considered eco-indicators of ocean health and may serve as an early warning for adverse effects on humans including exposure to industrial chemicals, and therefore their protection is an important component to mitigate the current global biodiversity crisis.^6^^,^^7^^,^^8^ Among the anthropogenic stressors affecting toothed whales, gillnet bycatch, being unintentional fishery catches, is a main driver behind the population declines of some species, and previous estimates suggest that hundreds of thousands of individual odontocetes are accidentally captured each year.^7^^,^^9^^,^^10^^,^^11^^,^^12^ Odontocetes often become entangled in fishing nets in both local artisanal fishery and large-scale open sea operations, leading to suffocation and drowning as they are unable to surface and breathe. Unfortunately, data on gillnet bycatch are often under-reported or entirely unrecorded, making it challenging to monitor and assess the impacts on local and regional populations, including those at risk of extinction.

Despite decades of efforts, the issue of odontocete gillnet bycatch requires renewed focus since significant progress remains lacking, even as hundreds of thousands of individuals continue to be caught yearly.^9^ Combined with overfishing, which can deplete prey availability, and bycatch from longline and trawling operations, gillnet bycatch poses severe threats to these long-lived and slow-reproducing species that invest heavily in their offspring. Urgent changes in fisheries management are desperately needed to decrease species loss and halt population declines globally.^7^^,^^13^ Altogether, this requires targeted mitigation of gillnet bycatch and additional anthropogenic impacts to protect these species.^14^^,^^15^^,^^16^

Information about odontocete gillnet bycatch is scattered and often varies by location, year, and species and is frequently outdated or confined to gray literature. Here, for the first time, we collect and synthesize data for a meta-analysis of global small- and large-scale gillnet bycatch, population estimates, and International Union for Conservation of Nature (IUCN) Red Listing conservation status to assess the risks faced by various toothed whale species inhabiting marine, estuarine, and riverine ecosystems between 1990 and 2020.

## Context

**Figure 2:**
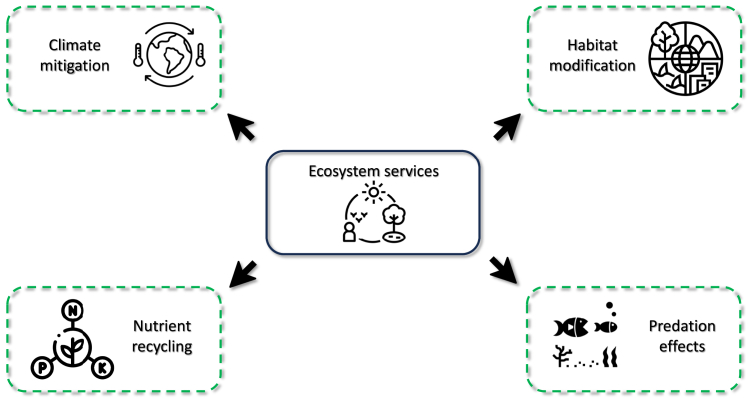
Ecosystem services of odontocetes include climate mitigation through sedimentation, nutrient recycling to and from deep sea and shallow waters, habitat modification, and top-down predation effects

A text-book example of odontocete gillnet bycatch is Mexico’s vaquita (*Phocoena sinus*), which is the world’s smallest and most threatened odontocete, with a total population size estimated in 2021 to be only 5–15 individuals.^17^^,^^18^ This population decline was not driven by inbreeding but rather by direct gillnet bycatch mortality, mainly from illegal, unreported, and unregulated artisanal gillnet fishing targeting totoaba fish—coincidentally another critically endangered species.^17^^,^^19^^,^^20^^,^^21^ These practices unfortunately still occur and impact vaquitas’ last habitat sanctuary in the upper reaches of the Gulf of California, even though the area is a protected United Nations Educational, Scientific and Cultural Organization (UNESCO) World Heritage site. In addition to gillnet bycatch and overfishing, other multiple environmental stressors including hunting, pollution, vessel traffic including underwater noise and disturbance affect the populations of odontocetes. Odontocetes are apex predators in marine ecosystems, and their overexploitation may directly and indirectly influence trophic cascades, marine communities, and population dynamics (Figure 2).^2^ These whales provide species- and ecosystem-specific top-down regulation of food webs including fish species, invertebrates, and other biota, helping to ensure efficient trophic transfer of nutrients and energy flow across ecosystems.^22^^,^^23^ Therefore, global odontocete gillnet bycatch has overarching impacts on wider marine ecosystem structure and functions.^2^ Collectively this means that odontocete gillnet bycatch significantly undermines the vital ecosystem services these species provide, including mitigation of climate change and vertical and horizontal nutrient mixing and cycling that is obviously important for the future of sustainable blue foods and economies, as well as in the context of planetary health.^1^^,^^24^

In addition to gillnet bycatch, other important anthropogenic factors to consider include both underwater noise, environmental pollution, hunting, and overfishing. Polychlorinated biphenyl pollution is considered a major driver in the reduction of killer whales populations,^14^ while underwater noise is believed to mask the ability of odontocetes to detect gillnets. Therefore, targeted efforts are needed to reduce environmental pollution and underwater noise, alongside advancing sustainable fisheries to minimize odontocetes gillnet bycatch.^25^ Lessons learned from pelagic gillnets targeting tuna in the Indian Ocean suggest that shifting from gillnets to longlines, when feasible, may reduce bycatch for some species. However, this shift could still negatively affect other species impacted by gillnet bycatch.^26^ Additionally, it may concomitantly increase tuna landings, demonstrating the socioeconomic complexity of this issue. This multi-faceted approach to ensure habitat refugia is a critical aspect of cetacean conservation biology in the context of gillnet bycatch.

## Meta-analyses

We provide the first global meta-analyses on small- and large-scale gillnet bycatch estimates of odontocetes compiling population size, gillnet bycatch, and conservation status leading to species-specific risk estimates during 1990–2020. The study includes dolphin and porpoise species inhabiting marine, estuarine, and riverine ecosystems. Such gillnet bycatch is largely under-reported, as it varies by year, species, and location. In the worst cases, it goes entirely unmonitored, resulting in a scarcity of systematic and harmonized data in the scientific literature. Therefore, information was somewhat inconsistent across taxa, and for some species only non-peer-reviewed, gray literature data were available for gillnet bycatch impacts. The literature search was conducted on the IUCN Red List of Threatened Species (https://www.iucnredlist.org/), PubMed, ScienceDirect, Google, Google Scholar, EBSCO, ProQuest, ScienceDirect, and MEDLINE including gray literature scientific reports combining the search terms “toothed whale OR odontocete” and/or “toothed whale OR cetacean” and/or “gillnet” and/or “bycatch.” The search was conducted from December 2, 2023, to June 10, 2024. We also did not distinguish between scientifically designed observer programs, marine mammal and wildlife stranding networks, reports from fishermen, or other expert consultations, as doing so would have significantly restricted the already limited and scarce available information. Also, there were no language restrictions applied to the literature review. We then used these data to compile all available information about population size and gillnet bycatch for a given period in each region categorized by each species, and then we synthesized this into yearly gillnet bycatch rate (%) and total gillnet bycatch (%) of population size along 1990–2020 (Table 1). We did this to extrapolate accumulated numbers for each period per species, yet only limited information was available for a limited period (years) for each species at each study location. We did not take population growth into account as this information was not available.

**Table 1:** Historic and current gillnet bycatch of 22 odontocetes and their roughly estimated numbers during1990–2020

Species/sub-species	Family	Latin name	Acronym	Region	IUCN status	Estimated population size	Estimated gillnet bycatch (period)	Yearly gillnet bycatch	Yearly gillnet bycatch rate (%)	%-total gillnet bycatch of population size	Reference
False killer whale	*Delphinidae*	*Pseudorca crassidens*	FKW	Taiwan, China, Hawaii	near threatened	<200	100 (2010–2020)	10	5	50.0	Baird RW et al.^27^
Irrawaddy dolphin	*Delphinidae*	*Orcaella brevirostris*	IRD	Asia	endangered	92	40 (2009–2011)	20	21.8	43.5	Temple AJ et al.^10^, WWF^28^ Whitty TS et al.^29^, Sonne C et al.^30^, Brownell et al.^31^ and Minton G et al.^32^
Burmeister’s porpoises	*Phocoenidae*	*Phocoena spinipinnis*	BUP	South America	near threatened	<500	205 (2002–2007)	29.3	8.2	41.0	Minton et al.^33^ Mangel JC et al.^34^ and Felix F et al.^35^
Vaquita	*Phocoenidae*	*Phocoena sinus*	VAQ	North America	critically endangered	30	8 (2016–2018)	4	13.3	26.7	Read AJ et al.^9^, Temple AJ et al.^10^, WWF^36^ Fisheries N et al.,^37^ and Gulland F et al.^38^
Guiana dolphin	*Delphinidae*	*Sotalia guianensis*	GUD	South America	near threatened	1,371–2,393	540 (2017–2019)	270	11-3-19.7	22.6–39.4	Temple et al.^10^, Domit^39^, Briceño et al.^40^ and Secchi et al.^41^
Common dolphin	*Delphinidae*	*Delphinus delphis*	COM	Mediterranean, Atlantic Ocean, Indian Ocean	least concern	1,000,000	200,000 (2000–2020)	10,000	2	20.0	Anderson et al.^12^, Mannocci et al.^42^, National park service^43^, Braulik^44^, and NOAAFisheries^45^
Striped dolphin	*Delphinidae*	*Stenella coeruleoalba*	STD	NE Atlantic, Pacific and Indian Ocean	least concern	2,000,000	400,000 (2000–2020)	20,000	2	20.0	Anderson et al.^12^, NOAAFisheries^45^, Group CM^46^, Ross^47^, and Braulik^48^
Common bottlenose dolphin	*Delphinidae*	*Tursiops truncatus*	BOD	global	least concern	600,000	100,000 (2000–2020)	5,000	0.83	16.7	Temple et al.^10^, Anderson et al.^12^, Mangel JC et al.^34^, NOAA Fisheries^45^, WWF^49^, Williams et al.^50^, and Hammond et al.^51^
Indo-Pacific bottlenose dolphin	*Delphinidae*	*Tursiops aduncus*	IP-BOD	Indo-Pacific	near threatened	33–35,000	20,000 (2000–2020)	1,000	2.9–3.03	57	Temple et al.^10^, Anderson et al.^12^, Mangel et al.^34^, WWF^49^, Williams et al.^50^, and Braulik et al.^52^
Spinner dolphin	*Delphinidae*	*Stenella longirostris*	SPD	global	least concern	>650,000	180,000 (2000–2020)	9,000	1.38	15.4	Anderson et al.^12^, ANIMALIA^53^, Lee et al.^54^, and Braulik^55^
Hector’s dolphin	*Delphinidae*	*Cephalorhynchus hectori*	HED	New Zealand	endangered	7,500	910 (2000–2006)	1512	2.02	12.1	Read et al^9^, Temple et al.^10^, WWF^56^, Slooten Slooten^57^, and Reeves^58^
Maui’s dolphin	*Delphinidae*	*Cephalorhynchus hectori* ssp. *maui*	MAD	New Zealand	critically endangered	57	5 (2012)	5	8.77	8.8	Temple^10^, Slooten^59^, IWC^60^, and Reeves et al.^61^
Harbor porpoise	*Phocoenidae*	*Phocoena phocoena*	HAP	North Atlantic & Pacific Ocean	least concern	700,000	51,996 (2006–2018)	4,333	0.62	8.5	Read et al.^9^, Temple et al.^10^, NOAA Fisheries^45^, Williams^50^, Moan et al.^62^, Larsen^63^, and Braulik^64^
Narrow-ridged finless porpoise	*Phocoenidae*	*Neophocaena asiaeorientalis*	NRF	West coast of Korean Peninsula	endangered	36,000	23,000 (2005–2011	3,833	10.6	64	–
Yangtze finless porpoise	*Phocoenidae*	*Neophocaena asiaeorientalis* ssp. *asiaeorientalis*	YFP	Asia	critically endangered	1,012	30 (2013–2014	30	1.48	2.96	Braulik et al.,^65^ Song K-J. et al.,^66^ Larson C et al.,^67^ Wang D et al.^68^
Commerson’s dolphin	*Delphinidae*	*Cephalorhynchus commersonii*	COD	South America	least concern	3,200	66 (1999–2000	66	1.03	2.0	Leatherwood et al.,^69^ Iñiguez MA et al.,^70^ and Crespo E et al.^71^
La Plata dolphin	*Pontoporiidae*	*Pontoporia blainvillei*	LPD	South America	vulnerable	40,000	517 (1999–2009	51.7	0.13	1.29	Temple AJ et al.,^10^, MARINEBIO^72^ Prado JH et al.,^73^ Zerbini AN et al.^74^
Indo-Pacific humpback dolphin	*Delphinidae*	*Sousa chinensis*	IPD	Asia	vulnerable	>2,000	13–16 (2000–2013	1–1.23	0.05–0.06	0.8	Temple AJ et al.,^10^ Guo L et al.,^75^ Liu M et al.,^76^ Collins T et al.,^77^ and Jefferson TA et al.^78^
Atlantic humpback dolphin	*Delphinidae*	*Sousa teuszii*	AHD	West Africa	critically endangered	<3,000	4 (2011–2014	1.33	0.04	0.13	Temple AJ et al.,^10^ Fisheries N et al.,^79^ Koen and van Waerebeek MU et al.^80^
Fraser’s dolphin	*Delphinidae*	*Lagenodelphis hosei*	FRD	global	least concern	289,000	63 (2017–2018	63	0.01	0.02	Fisheries N et al.,^81^ Obienu J et al.,^82^ and Kiszka JB et al.^83^
Risso’s dolphin	*Delphinidae*	*Grampus griseus*	RID	North America	Least concern	>315,000	74 (1990–2019	2.55	0.001	0.02	Mangel JC et al.,^34^, ANIMALIA^84^ Amir OA et al.,^85^ and Lanfredi C et al.^86^
Cuvier’s beaked whale	*Ziphiidae*	*Ziphius cavirostris*	CBW	North America	least concern	>100,000	–	–	–	–	Williams R et al.^50^, ANIMALIA^87^ and Baird RW et al.^88^

-: unknown. Yearly gillnet bycatch = estimated gillnet bycatch/years in period. Yearly gillnet bycatch rate (%) = (yearly gillnet bycatch/estimated population size) × 100. %-total gillnet bycatch of population size = (estimated gillnet bycatch/estimated population size) × 100.

## Global gillnet bycatch patterns

**Figure 3:**
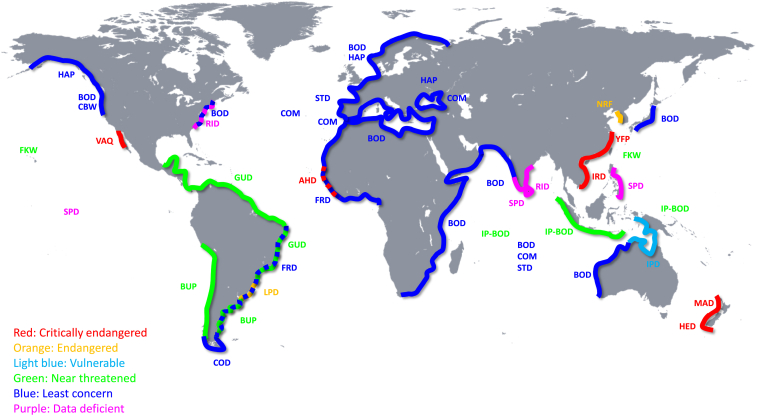
Gillnet bycatch localities of 22 odontocetes and their global IUCN Red List conservation status during 1990–2020 AHD, Atlantic humpback dolphin; BOD, common bottlenose dolphin; BUP, Burmeister’s porpoise; CBW, Cuvier’s beaked whale; COD, Commerson’s dolphin; COM, common dolphin; FKW, false killer whale; FRD, Fraser’s dolphin; GUD, Guiana dolphin; HAP, harbor porpoise; HED, Hector’s dolphin; IP-BOD, Indo-Pacific bottlenose dolphin; IPD, Indo-Pacific humpback dolphin; IRD, Irrawaddy dolphin; LPD, La Plata dolphin; MAD, Maui’s dolphin; NRF, narrow-ridged finless porpoise; RID, Risso’s dolphin; SPD, spinner dolphin; STD, striped dolphin; VAQ: vaquita; YFP, Yangtze finless porpoise.

Figure 3 shows global gillnet bycatch of vaquita and 21 other odontocetes including their IUCN Red List conservation status. Global gillnet bycatch numbers are estimates; however, it appears that it is the greatest in Asia, East Africa, and South America accounting for approximately 50,000 individuals annually during 1990–2020 (Table 1). The number of odontocetes is also decreasing in Asia, including the Yangtze finless porpoise (*Neophocaena asiaeorientalis*) and the Irrawaddy and Ganges River dolphins. These three species all experience severe population declines from gillnet bycatch, and from a lack of suitable habitats for feeding and reproduction. This has driven the population of Yangtze finless porpoises down to ∼250 individuals, while the Irrawaddy River dolphin population has declined to only ∼3,000 individuals, largely because of gillnet-related mortality.^30^^,^^31^^,^^67^ Likewise, Franciscana (*Pontoporia blainvillei*) and Guiana dolphin (*Sotalia guianensis*) populations are also affected by gillnet mortalities along the east coast of South America,^26^ making the Franciscana dolphin the most endangered small cetacean in the western region of the South Atlantic Ocean. The Maui dolphin (*Cephalorhynchus hectori maui*) populations are classified as critically endangered with a gillnet bycatch rate of 8.8% in New Zealand (Table 1). Gillnet bycatch-related mortalities are also significant in the Indian Ocean and are often linked to large-scale, industrial tuna fishing operations.^12^ In particular, gillnets used for pelagic fish, such as tuna species, result in significant gillnet bycatch of dolphins including striped dolphin (*Stenella coeruleoalba*), common dolphin (*Delphinus delphis*), spinner dolphin (*Stenella longirostris*), and Indo-Pacific bottlenose dolphin (*Tursiops aduncus*) with lower mean estimate population-size-weighted annual gillnet bycatch estimates of 20,000, 10,000, 9,000, and 1,000 individuals, respectively, during 2000–2020.^12^

Comparatively, the estimated numbers of dolphin gillnet bycatch-related mortalities by tuna fisheries in the Atlantic Ocean and Mediterranean Sea are several magnitudes lower, possibly due to greater use of longline fishing compared to gillnets.^26^ In Europe, harbor porpoise annual gillnet bycatch was estimated to be 4,333 individuals during 2006–2018 covering North Sea, Norwegian coast, Skagerrak, Kattegat, Belt Seas, and the Western Baltic Sea (Table 1). Annual gillnet bycatch also affects harbor porpoises in the US and Canada and is estimated to be ∼600 individuals per year in the same period highlighting the widespread and global nature of this problem (Table 1). In Europe, annual gillnet bycatch of harbor porpoises is estimated to be >2,700, accounting for 2% of the population found across European waters. Similar patterns of gillnet bycatch also affect harbor porpoises in British Columbia, highlighting the global nature of the problem.

**Figure 4:**
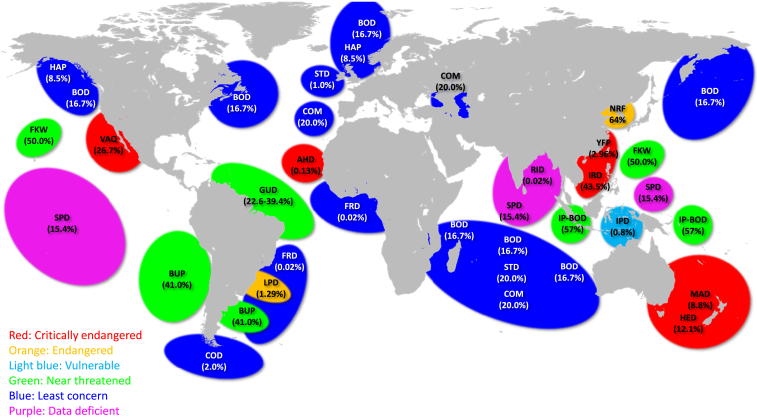
Regions of gillnet bycatch and % of population size of 22 odontocetes during 1990–2020 based on the IUCN Red List Conservation Status and estimated gillnet bycatch numbers AHD, Atlantic humpback dolphin; BOD, common bottlenose dolphin; BUP, Burmeister’s porpoise; CBW, Cuvier’s beaked whale; COD, Commerson’s dolphin; COM, common dolphin; FKW, false killer whale; FRD, Fraser’s dolphin; GUD, Guiana dolphin; HAP, harbor porpoise; HED, Hector’s dolphin; IP-BOD, Indo-Pacific bottlenose dolphin; IPD, Indo-Pacific humpback dolphin; IRD, Irrawaddy dolphin; LPD, La Plata dolphin; MAD, Maui’s dolphin; NRF, narrow-ridged finless porpoise; RID, Risso’s dolphin; SPD, spinner dolphin; STD, striped dolphin; VAQ: vaquita; YFP, Yangtze finless porpoise.

Of the species IUCN Red Listed as being vulnerable (*n* = 2), endangered (*n* = 3), or critically endangered (*n* = 5), five are at intermediate or high concern from population impacts of gillnet fisheries, which appears to be most pronounced in the Global South (Table 1 and Figure 4). In addition, species that are IUCN Red Listed as being of least concern such as common bottlenose dolphin, common dolphin, and striped dolphin are by-caught to an extent that also raises concerns and should be considered when Red Listed. These patterns of global gillnet bycatch of several odontocete populations suggest the need for increased attention, visibility, and immediate conservation action to significantly reduce mortalities, and to address population declines at a global scale.

The global threats to toothed whales from gillnet bycatch are a result of both industrial and small-scale fisheries (SSFs).^89^^,^^90^^,^^91^ The SSFs compose the majority (i.e., 80% in the European Union [EU]) of global fishing fleets.^92^ This issue is a considerable challenge as SSF is largely considered to be more sustainable compared to large industrial fishing practices that often catch non-targeted species and therefore needs to be urgently addressed as it poses significant conservation concerns.^89^^,^^90^^,^^91^ These SSFs and artisanal fisheries are regionally diverse throughout the world’s ocean basins. This makes it difficult to classify and quantify, on a global scale, how these practices are in direct conflict with the economy of rural societies of developing countries.^67^ This anthropogenic threat from gillnet bycatch therefore requires meaningful protective measures and environmental governance strategies to avoid further reductions of marine mammal populations and loss of species diversity, and to protect estuarine and marine ecosystem services and ocean health.

Aside from improving and rebuilding global fisheries in a more sustainable way, there is also a need for intergovernmental panels and regional collaboration to support this important conservation initiative.^13^ For example, in the western North Atlantic, there is currently much attention from federal, state, and regional fisheries management entities and non-governmental organizations (NGOs) to reduce gillnet bycatch of odontocetes. The closure of marine areas, enforced restrictions, seasonal shifts in fisheries, better gillnet bycatch assessments, and changes in fishing gear technology, including mesh size, have been instrumental in achieving gillnet bycatch reduction and acoustical deterrents (pingers).^93^

## Gillnet bycatch mitigation measures

Local subsistence and indigenous communities must retain fish harvesting rights in local rivers and marine waters. This effort avoids poaching and illegal fishery activities as identified by the United Nations (UN) Sustainable Development Goals (SDGs) and creates financial income and benefits, community development, and sustainable eco-tourism. Decision-makers need to better integrate cultural, economic, and social aspects of sustainable fisheries to reduce global cetacean gillnet bycatch occurrence, frequency, and severity. A proposed, suitable approach is to establish local management boards of resource users and related entities where steering groups have a broad membership from the local community to evaluate the program’s effectiveness. In addition, adaptive management should include multiple stakeholders, research institutions, NGOs, and local government representatives to ensure that necessary support and aid are available, including funding, relevant expertise, and critical infrastructure. These actions may help to ensure better alliances and collaboration between local fishermen, researchers, and NGOs within an ecological framework that could also include tourism as financial and conservation incentive components. Such approaches may serve as an effective model for other taxa dealing with similar threats and population declines. Therefore, identifying the most appropriate mitigation measures requires an understanding of the nature of the interactions based on collaborations between fishers, fisheries managers, marine mammal experts, and fisheries engineers. These are all contributing collective, critical expertise for developing, evaluating, and implementing gillnet bycatch reduction measures.

There are several ways to try to reduce the gillnet bycatch of odontocetes as outlined in the Gillnet Bycatch Management Information System (https://www.bmis-gillnetbycatch.org/mitigation-techniques). Depending on the region and species, it is necessary to apply different methods in, e.g., river ecosystems, along shorelines, and at open ocean gillnet fisheries. Overall, this includes spatial closures (dynamic or real-time), acoustic alerting or deterrent devices, modifications to fishing gear, changes to fishing operations, and other strategies.^94^ For example, managing gillnet bycatch of river dolphins in Asia, such as the Irrawaddy dolphin and Yangtze finless porpoise, may require implementing spatial closures in their known breeding and feeding habitats. This can be potentially achieved by using acoustic deterrent devices or mitigation of fishing gear such as weak links with lower breaking strength, since the water visibility may prohibit the efficiency of, for example, changing the gillnet color and luminosity.^94^ It must however be noted that acoustic deterrent devices are not that simple to use as they may also attract the animals, and individuals may adapt or in severe cases hearing injuries may occur.^94^ Examples of mitigation strategies are listed in Table 2.

**Table 2:** Examples of mitigation measures of gillnet fishery gillnet bycatch of odontocetes

Habitat	Species	Measures	Cons
River ecosystems	Irrawaddy dolphin Yangtze finless porpoise	spatial closures acoustic deterrent weak links gillnet color/luminosity satellite telemetry/home range local indigenous knowledge	reduced local food security habituation to acoustic deterrents hearing injury logistically impossible economic losses research budgets inaccurate information
Along shorelines	harbor porpoise Hector’s dolphin Vaquita	spatial closures permanent sanctuaries pound nets	economic losses economic incentives work labor
Open ocean	common dolphin striped dolphin bottlenose dolphin	longlines (hooks) colored gillnets diurnal regulation smaller net size electric barriers	investments economic losses reduced food security

In open ocean habitats, where gillnet bycatch of common, striped, and bottlenose dolphins is high, other measures, such as colored gillnets, fishing within specific diurnal rhythms, smaller gillnet size, and electric barriers, may be required to reduce impacts.^94^ In bays and fjords, and along coastlines, closures mostly work for species not being threatened as it is not logistically possible, nor practical, to close the fishery in these large areas. In specific cases, a targeted and complete stop of gillnet fishery is required for vaquita, and Hector’s dolphin, as even permanent spatial closures are not adequate and sufficient to save these species. Spatial closures to restrict gillnet fishery have been established in Australia, New Zealand, Mexico, and the USA to protect harbor porpoise (*Phocoena phocoena*), supported by collaboration with individual fishermen during specific time periods by providing incentives.^94^ Such collaborations may be applied successfully to other coastal and offshore areas of the Indian, Atlantic, and Pacific Oceans combined with permanent sanctuaries.

Management of cetaceans will benefit from local, ecological knowledge and scientific expertise, including critical guidance from indigenous communities. It is impossible to protect these marine mammal populations without identifying and understanding odontocetes feeding ecology, reproductive dynamics, and nursery habitats. Satellite telemetry can be a valuable tool for mapping core areas and home ranges to sustain and facilitate successful reproduction and population viability. Additionally, other technology has been applied to cetaceans with mixed results. Among these are local fishermen’s’ use of pingers and reflective gillnets outside refugia to reduce gillnet bycatch and achieve ecosystem balance.^95^ In addition, studies suggest that time of day is a critical parameter to consider when mitigating gillnet bycatch in the open Atlantic Ocean and that this may also be applied to SSFs elsewhere. For some species, the probability of gillnet bycatch is higher during crepuscular time periods, partly due to diurnal movements.^96^ Substituting gillnet fisheries by passive stationary pound nets may alleviate some problems for regional fisheries, where this type of gear has been effective. A key limiting factor in this proposed strategy is that any stoppage in fisheries operations challenges economic losses and food security in several developing countries. This makes unifying international support by international conservation boards such as the International Whaling Commission (IWC) complex and challenging.

## Outlook and research gaps

There are several regional and global organizations that could help to facilitate better management of global odontocetes gillnet bycatch. In the EU, for example, the International Council for the Exploration of the Sea gillnet bycatch working group advises how to quantify the impact of fisheries on odontocetes as reflected in the EU Council regulation 2019/1241. The IWC has endorsed a gillnet Bycatch Mitigation Initiative to promote effective gillnet bycatch prevention and collaborated with Food and Agriculture Organization of the UN to develop advice mainly for fishery managers, fishers, and researchers.^97^ Other expert groups including Agreement on the Conservation of Cetaceans of the Black Sea, Mediterranean Sea and contiguous Atlantic area (ACCOBAMS) and Agreement on the Conservation of Small Cetaceans of the Baltic and North Seas (ASCOBANS) may also help to mitigate gillnet bycatch in Europe, despite the fact that not all adapted measures are sufficient.^98^

Management solutions need to be identified and enforced at region-specific scales to meet the needs of successful, adaptive management strategies for gillnet bycatch. These implementations have increased political support and public awareness of coastal and aquatic resource management and in conjunction with the Global Whale Entanglement Response Network help support relevant UN SDGs and biodiversity initiatives that are considered critical for gillnet bycatch mitigation. In addition, the impacts of fishing gillnet bycatch in protected areas need to be more properly addressed and prioritized by natural resource managers, policy makers, and biodiversity and conservation agencies to support these initiatives.

Odontocetes are known to migrate, and so multi-national collaboration is required to manage the populations through joined collaboration such as the IWC, Convention on the Conservation of Migratory Species of Wild Animals, and Convention on International Trade in Endangered Species of Wild Fauna and Flora to put on pressure on those countries that have so far failed their fisheries management to reduce risk of gillnet bycatch and extinction.^98^^,^^99^^,^^100^ In addition, there need to be improvements in the current strategies supported by new research methods on how to monitor and assess what is needed to protect odontocetes from fishery gillnet bycatch. Evaluating the use of the US Marine Mammal Protection Act to see if economic sanctions and incentives could provide the necessary motivation to protect odontocetes from gillnet bycatch and avoid further loss of biodiversity and ecosystem services could be further explored.

## Limitations of the study

Our investigation has a few limitations that need to be considered and that are common to many other studies conducting global scale meta-analyses. First, information about gillnet bycatch is scattered throughout the scientific and gray literature, making it difficult to obtain harmonized data to assess region- and species-specific bycatch. Furthermore, up-to-date information on both gillnet bycatch and population sizes is lacking, and those that do exist often have large uncertainties in historic and contemporary datasets, making it difficult to highlight new findings. Regardless of these limitations, the findings obtained in this study can be used to inform local and global authorities about what measures are needed to reduce odontocete gillnet bycatch to sustain the species and the ecosystem services they provide.

## Acknowledgments

We acknowledge Dr. Doug Adams for comments on initial drafts of this Perspective. Su Shiung Lam would like to thank Chandigarh University for the support provided.

## Declaration of interests

The authors declare no competing interests.
